# Microbial Successions and Metabolite Changes during Fermentation of Salted Shrimp (Saeu-Jeot) with Different Salt Concentrations

**DOI:** 10.1371/journal.pone.0090115

**Published:** 2014-02-28

**Authors:** Se Hee Lee, Ji Young Jung, Che Ok Jeon

**Affiliations:** Department of Life Science, Research Center for Biomolecules and Biosystems, Chung-Ang University, Seoul, Republic of Korea; Wageningen University, Netherlands

## Abstract

To investigate the effects of salt concentration on saeu-jeot (salted shrimp) fermentation, four sets of saeu-jeot samples with 20%, 24%, 28%, and 32% salt concentrations were prepared, and the pH, bacterial and archaeal abundances, bacterial communities, and metabolites were monitored during the entire fermentation period. Quantitative PCR showed that *Bacteria* were much more abundant than *Archaea* in all saeu-jeot samples, suggesting that bacterial populations play more important roles than archaeal populations even in highly salted samples. Community analysis indicated that *Vibrio*, *Photobacterium*, *Psychrobacter*, *Pseudoalteromonas*, and *Enterovibrio* were identified as the initially dominant genera, and the bacterial successions were significantly different depending on the salt concentration. During the early fermentation period, *Salinivibrio* predominated in the 20% salted samples, whereas *Staphylococcus*, *Halomonas*, and *Salimicrobium* predominated in the 24% salted samples; eventually, *Halanaerobium* predominated in the 20% and 24% salted samples. The initially dominant genera gradually decreased as the fermentation progressed in the 28% and 32% salted samples, and eventually *Salimicrobium* became predominant in the 28% salted samples. However, the initially dominant genera still remained until the end of fermentation in the 32% salted samples. Metabolite analysis showed that the amino acid profile and the initial glycerol increase were similar in all saeu-jeot samples regardless of the salt concentration. After 30–80 days of fermentation, the levels of acetate, butyrate, and methylamines in the 20% and 24% salted samples increased with the growth of *Halanaerobium*, even though the amino acid concentrations steadily increased until approximately 80–107 days of fermentation. This study suggests that a range of 24–28% salt concentration in saeu-jeot fermentation is appropriate for the production of safe and tasty saeu-jeot.

## Introduction

Jeotgal, Korean traditional fermented seafood, is made by the fermentation of sea animals such as shrimp, anchovy, pollack intestines, corvina, and squid under high salt conditions. Jeotgal is consumed as fermented seafood by itself or as an additive to other foods such as kimchi to improve the taste (flavor) or fermentation efficiency. Processing of jeotgal by fermentation without the sterilization of raw materials leads to the growth of various microorganisms, derived from the raw materials, during fermentation [1], [2]. It has been generally accepted that diverse microorganisms, including *Bacteria* as well as *Archaea* are involved in jeotgal fermentation because jeotgal is fermented under highly salted conditions, [3] and many studies have been performed to isolate *Bacteria* and *Archaea* to understand jeotgal fermentation processes [4]–[6]. At the time of writing, more than 25 novel bacterial and archaeal species have been isolated from jeotgal (http://bacterio.net) [7]. However, it has been recently reported that *Archaea* may not play important roles in jeotgal fermentation [8], [9].

Culture-based approaches have been widely applied to community analysis of fermented seafood [5]–[11], but these studies have produced a limited amount of information because of the presence of many unculturable microorganisms [3]. Therefore, culture-independent approaches such as denaturing gradient gel electrophoresis (DGGE) and 16S rRNA gene clone library have been used as alternatives to analyze microbial communities of fermented seafood including many unculturable microorganisms [12]–[14], but they also have limitations because they include laborious procedure steps and produce a low depth of information. To overcome some of these limitations, pyrosequencing has been broadly used to investigate microbial communities of fermented food [3], [15]–[20]. It has also been reported that microorganisms as well as diverse endogenous enzymes such as proteinases and lipases derived from the fish itself contribute to seafood fermentation [9], [20]–[22]. Because metabolite production is the result of microbial communities and fish-derived enzymes [23], studies of microbial communities and metabolites are indispensable for obtaining a better understanding of seafood fermentation processes [23]. Metabolite analysis using proton nuclear magnetic resonance (^1^H NMR) spectroscopy is a comprehensive and easy technique for simultaneously monitoring diverse metabolites in fermented food processes [24]. A combination of pyrosequencing and ^1^H NMR has been suggested to be one of the best ways to better understand the relationships between bacterial successions and metabolite changes during food fermentation [8], [25]–[29].

Saeu-jeot, made by fermentation of tiny shrimp (*Acetes japonicus*), is the most representative and best-selling jeotgal in Korea. Saeu-jeot fermentation is processed under uncontrolled conditions without the sterilization of raw materials, which may cause the growth (or survival) of pathogens or putrefaction during fermentation. Therefore, saeu-jeot is generally fermented at low temperatures under highly salted conditions to prevent pathogenic growth and putrefaction. It has been generally thought that temperature and salt concentration are the most important factors to affect microbial growth and enzyme activity and low temperatures and high salt conditions lead to a long saeu-jeot fermentation time [30]–[32]. Many Korean companies have taken an interest in increasing fermentation temperature and reducing salt concentration to produce saeu- jeot economically. In the previous paper [9], the effects of fermentation temperature on saeu-jeot fermentation were investigated, but until now the effects of salt concentration on microbial successions and metabolite changes during saeu-jeot fermentation have not yet been explored. Therefore, the main objective of this study was to investigate the effects of different salt concentrates on saeu-jeot fermentation by investigating microbial successions and metabolite changes. This study may provide a greater understanding of saeu-jeot fermentation processes for the economical production of safe and high quality saeu-jeot.

## Materials and Methods

### Ethic Statement

A field test was not performed in this study. Salted saeu-jeot samples were prepared in the laboratory using shrimp that was bought from a market near the Yellow Sea in Korea. No specific permissions were required for this study because the shrimp was not protected or endangered species.

### Preparation of saeu-jeot samples with different salt concentrations

Four sets of saeu-jeot samples with different salt concentrations [approximately 20%, 24%, 28%, and 32% (w/v)] were prepared in triplicate using shrimp (*A*. *japonicas*) as described previously [8], [9]. Briefly, fresh tiny shrimp (about 4–6 cm in length) caught from the Yellow Sea in South Korea were equally dispensed into twelve plastic containers to include 1.5 kg shrimp and 307 g, 381 g, 461 g, and 548 g of solar salts (Shinan, Korea), and 600 ml of 20%, 24%, 28%, and 32% (w/v) solar salt solution were added to each container in triplicate, respectively. The four sets of saeu-jeot samples were incubated at 15°C. Four milliliters of saeu-jeot soups (liquid parts of saeu-jeot) were intermittently sampled from the twelve containers, and their pH values were measured. Large particles from the saeu-jeot soups were removed by filtration using four layers of sterile coarse gauze (Daehan, Korea), and microorganisms were harvested from the filtrates by centrifugation (8,000 rpm for 20 min at 4°C). Microorganisms harvested from three containers with the same salt concentration were combined and stored at −80°C for bacterial community analysis, but the supernatants were stored separately at −80°C for respective metabolite analyses. Two milliliters of saeu-jeot soups were also sampled and centrifuged for measuring 16S rRNA gene copies using quantitative real-time PCR (qPCR). The NaCl concentrations of the saeu-jeot samples with different salt concentrations were measured by titration with silver nitrate according to the Mohr method [33].

### qPCR

The abundance of *Bacteria* and *Archaea* in the saeu-jeot samples was estimated using qPCR according the method described previously with some modifications [8]. Briefly, 100 ng of salmon testes DNA (Sigma) was added as an exogenous and internal standard into the pellet derived from 2.0 ml of saeu-jeot soup, and the total genomic DNA was then extracted using a FastDNA Spin kit (MPbio, USA) according to the manufacturer's instructions. To measure the total number of 16S rRNA gene copies in *Bacteria* and *Archaea*, two qPCR primer sets, bac340F/bac758R and arch109F/arch344R [34], [35], respectively, were used. Sample-to-sample variations caused by different genomic DNA recoveries and PCR amplification efficiencies were normalized on the basis of qPCR results using the primer set, Sketa2-F (5′-GGT TTC CGC AGC TGG G-3′)/Sketa2-R (5′-CCG AGC CGT CCT GGT CTA-3′), targeting the internal transcribed spacer region 2 of the rRNA gene operon in salmon testes DNA, as described previously [36]. The qPCR amplifications were conducted as described previously [25]. Two standard curves for the calculations of the bacterial and archaeal 16S rRNA gene copies were generated using pCR2.1 vectors (Invitrogen, USA) carrying bacterial (*Salimicrobium*) and archaeal (*Halarchaeum*) 16S rRNA genes derived from a saeu-jeot sample [8].

### PCR amplification for barcoded pyrosequencing

Total genomic DNA of the combined pellets from the triplicate samples was extracted using the Fast-DNA Spin kit (MPbio) according to the instructions of the manufacturer. Bacterial 16S rRNA genes containing hypervariable regions were amplified using the universal primer set, Bac27F (5′-adaptor B-AC-GAG TTT GAT CMT GGC TCA G-3′)/Bac541R (5′-adaptor A-X-AC-WTT ACC GCG GCT GCT GG-3′), where X denotes unique 7∼11 barcode sequences inserted between the 454 Life Sciences adaptor A sequence and the common linker, AC (Table S1). All PCR amplifications were performed as described previously [37], and the PCR products were purified using a PCR purification kit (Bioneer, Korea). The purified PCR products were quantified using a SynergyMx ELISA reader equipped with a Take3 multivolume plate (BioTek, USA), and a composite sample for pyrosequencing was prepared by pooling equal amounts of the purified PCR products.

### Pyrosequencing and data analysis

Pyrosequencing of the pooled sample was performed using a 454 GS-FLX Titanium instrument (Roche, Germany) at Macrogen (Korea). Sequencing reads generated from the pyrosequencing were processed using the RDP pyrosequencing pipeline (http://pyro.cme.msu.edu/)[38]. Sequencing reads were grouped by saeu-jeot sample based on their unique barcodes, and the barcodes were then removed. Sequencing reads with more than two ‘N’ (undetermined nucleotide), shorter than 300 bp read length, or average quality values below 20 (error rate 0.01) were excluded from further analysis. Putative chimeric reads were removed by the chimera.slayer command within the MOTHUR program [39]. The numbers of the high quality reads were normalized to the lowest number of reads (844 reads) by randomly deleting reads from the sequencing fasta files using a perl script called *selector.pl* [41]. The original and normalized sequencing reads were aligned using the RDP pyrosequencing aligner and the resulting aligned sequences were clustered at a 3% dissimilarity level using the complete-linkage clustering tool. Rarefaction curves [40] from the original sequencing reads were generated by the RDP pyrosequencing pipeline. Operational taxonomic units (OTU), Shannon-Weaver [41] and Chao1 indices [42], and evenness from the original and normalized sequencing reads were calculated using the RDP pyrosequencing pipeline.

To compare bacterial successions in saeu-jeot samples with different salt concentrations, taxonomic assignments of the bacterial high quality reads were performed at the phylum and genus levels using the RDP naïve Bayesian rRNA Classifier 2.5 trained on 16S rRNA training set 9 [43] at an 80% confidence threshold. The bacterial successions were confirmed using principal coordinate analysis (PCoA) based on representative sequences derived from the respective saeu-jeot samples. Briefly, the representative sequences were selected using CD-HIT [44], with an identity cutoff of 97%, and aligned using the RDP pyrosequencing pipeline based on the Silva database (v102). A neighbor-joining tree was constructed using the Kimura two-parameter model [45] within the PHYLIP software (ver. 3.68) and the NEXUS tree file was used as an input file for the weighted PCoA. The weighted PCoA was also performed using the sequencing data sets before and after removing singletons as described previously [46].

### Metabolite analysis using ^1^H NMR and statistical redundancy analysis

The profiles of the metabolites, including amino acids, monosaccharides, organic acids, and methylamines, during the saeu-jeot fermentation were analyzed using a Varian Inova 600-MHz NMR spectrometer (Varian, USA) according to a method described previously [8]. Identification and quantification of individual metabolites from the ^1^H NMR spectra were performed using the Chenomx NMR suite program (ver. 6.1, Chenomx, Canada) with 2,2-dimethyl-2-silapentane-5-sulfonate (DSS) as the internal standard. To investigate the metabolite changes and their correlations with bacterial abundances during saeu-jeot fermentation, statistical redundancy analysis (RDA) was performed using the vegan package [47] in the R programming environment (http://cran.r-project.org/) on the basis of all metabolites annotated from the ^1^H NMR spectra and the bacterial abundances in saeu-jeot samples with different salt concentrations, which was plotted as a triplot.

### Nucleotide sequence accession number

The pyrosequencing data of the 16S rRNA genes are publicly available in the NCBI Short Read Archive (SRA) under accession no. SRA039814.

## Results

### General features of saeu-jeot fermentation

**Figure 1:**
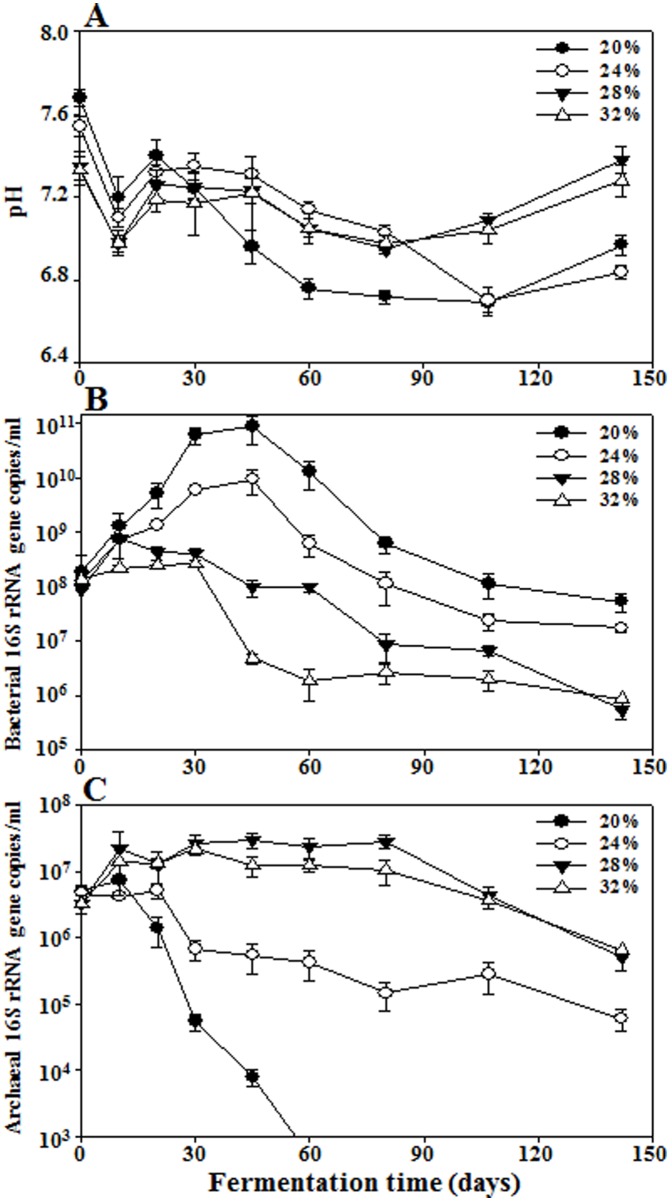
Profiles of pH (A) and bacterial (B) and archaeal (C) 16S rRNA gene copies in saeu-jeot samples with 20%, 24%, 28%, and 32% salt concentrations during saeu-jeot fermentation. Measurements of pH values and bacterial and archaeal 16S rRNA gene copies were performed in triplicate and the error bars represent the standard deviations.

Four sets of saeu-jeot samples with approximately 20%, 24%, 28%, and 32% (w/v) salt concentrations were incubated at 15°C for 142 days, and the NaCl concentrations were maintained at 19.9±1.1%, 24.7±0.3%, 28.1±1.1%, and 32.1±1.3% (w/v), respectively. The initial pH values of the saeu-jeot samples were approximately 7.3–7.7 (Fig. 1A). After the slight decrease of the pH values during the early fermentation period (day 10), the pH values increased a little for all saeu-jeot samples. After 20 days of fermentation, the pH values of the 20% and 24% salted samples decreased again to approximately pH 6.7, whereas those of the 28% and 32% salted samples were relatively constant.

A qPCR approach was used to estimate the abundance of *Bacteria* and *Archaea* in saeu-jeot samples. The initial bacterial 16S rRNA gene copies were approximately 1.3×10^8^ copies/ml. The bacterial 16S rRNA gene copies increased during the early fermentation period in all saeu-jeot samples (Fig. 1B), but the increases were much greater in saeu-jeot samples with lower salt concentrations. The 16S rRNA gene copies of the 20% and 24% salted samples increased to the highest values of ∼8.8×10^10^ and 9.5×10^9^ copies/ml, respectively, at 45 days, while those of the 28% and 32% salted samples only increased to ∼7.8×10^8^ and 2.6×10^8^ copies/ml, respectively. The initial 16S rRNA gene copies of *Archaea* were approximately 4.0×10^6^ copies/ml. The archaeal 16S rRNA gene copies of the 28% and 32% salted samples increased during the early fermentation period although the increases were not large, whereas those of the 20% and 24% salted samples decreased rapidly with a short lag time (Fig. 1C). The archaeal 16S rRNA gene copy number was much lower than the bacterial 16S rRNA gene copy number in the 20% and 24% salted samples during the entire fermentation period, as reported previously [8], [9]. The 16S rRNA gene copies of *Bacteria* were higher than those of *Archaea* in the 28% and 32% salted samples during the early fermentation period although the copy number of *Archaea* exceeded that of *Bacteria* during the late fermentation period.

### Bacterial diversity in saeu-jeot samples with different salt concentrations

**Figure 2:**
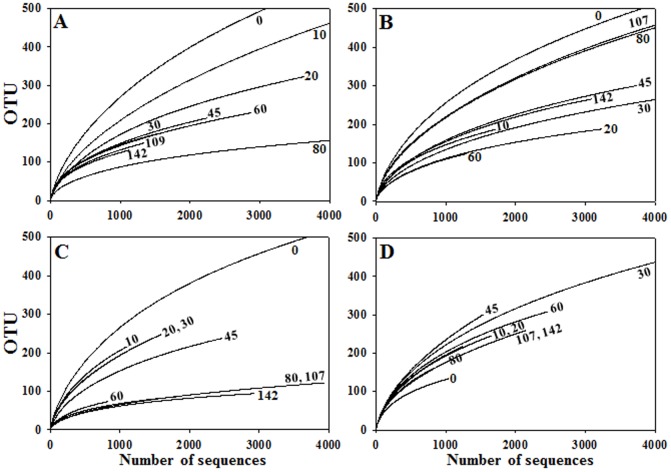
Rarefaction analysis of 16S rRNA gene sequences for the analysis of bacterial diversity in saeu-jeot samples with 20% (A), 24% (B), 28% (C), and 32% (D) salt concentrations during saeu-jeot fermentation. The rarefaction curves were constructed by the RDP pipeline at a 97% identity cutoff. The x- and y-axes indicate the numbers of sequencing reads sampled and the cumulative numbers of OTU (operational taxonomic units) recorded, respectively. Numbers beside the curves represent the fermentation time (days).

A total of 138,678 sequencing reads were obtained from barcoded pyrosequencing of 36 PCR amplicons for bacterial 16S rRNA genes. After trimming of the barcoded PCR primers and removal of the low-quality and chimera reads, a total of 102,571 high quality reads were obtained, with more than 473 bp average read length and an average of approximately 2,849 reads per sample. The rarefaction curves and microbial diversity indices were generated statistically for each sample (Fig. 2 and Table 1). The rarefaction curves showed that the bacterial diversities in the 20% and 24% salted samples decreased during the early fermentation period (the decrease was faster in the 24% salted samples than in the 20% salted samples) and the diversities increased during the late fermentation period, whereas in the 28% salted samples the bacterial diversities were relatively constant during the early fermentation period and the diversities decreased continually until the late fermentation period. In the 32% salted samples, the bacterial diversities of the samples fermented were higher than those of the initial samples, indicating no evident bacterial growth. The OTU, Shannon-Weaver diversity estimates, and Chao1 nonparametric richness estimator (Table 1) also supported the results of the rarefaction curve analysis that bacterial community changes were different depending on the salt concentrations of the saeu-jeot samples.

**Table 1:** Summary of the barcoded pyrosequencing data sets derived from the saeu-jeot samples and statistical analysis of microbial diversity.

Subject		Total reads	High quality reads	Average read length	OTU*	Shannon-Weaver*	Chao1*	Evenness*
Salt conc.	Days							
	0	6866	4662	477	610 (177)	4.9 (4.3)	1013 (282)	0.76 (0.82)
	10	12766	9353	490	714 (143)	4.3 (3.7)	1236 (358)	0.65 (0.74)
	20	6231	3649	477	323 (119)	3.8 (3.2)	557 (227)	0.66 (0.67)
	30	2170	1536	461	174 (125)	4.1 (3.8)	293 (201)	0.80 (0.80)
20%	45	2903	2249	439	215 (95)	4.1 (3.6)	333 (165)	0.76 (0.79)
	60	3743	2892	435	229 (94)	4.1 (3.6)	397 (147)	0.76 (0.79)
	80	6033	4328	476	161 (53)	2.9 (2.0)	251 (69)	0.56 (0.50)
	107	1712	1348	442	150 (107)	3.9 (3.7)	244 (157)	0.78 (0.79)
	142	1424	1129	432	131 (88)	3.9 (3.5)	193 (131)	0.80 (0.78)
	0	5653	4105	477	518 (161)	4.8 (4.1)	918 (251)	0.77 (0.81)
	10	2206	1718	484	187 (129)	3.5 (3.3)	295 (195)	0.66 (0.68)
	20	4817	3252	474	188 (88)	3.2 (3.2)	300 (141)	0.61 (0.71)
	30	6210	4564	473	280 (86)	3.5 (3.0)	444 (168)	0.62 (0.67)
24%	45	5129	3755	476	301 (99)	3.8 (3.1)	459 (135)	0.67 (0.67)
	60	1732	1307	477	124 (94)	3.3 (3.6)	185 (147)	0.68 (0.79)
	80	7700	5418	453	521 (143)	4.6 (3.9)	917 (247)	0.74 (0.78)
	107	7228	5344	438	524 (145)	4.8 (4.1)	820 (226)	0.76 (0.82)
	142	4108	3107	431	266 (109)	4.2 (3.6)	373 (232)	0.75 (0.76)
	0	9769	6638	482	630 (172)	5.0 (4.2)	885 (302)	0.77 (0.82)
	10	1432	1101	481	216 (180)	4.2 (4.1)	355 (323)	0.79 (0.80)
	20	1914	1598	479	248 (163)	4.3 (3.9)	487 (279)	0.77 (0.78)
	30	1062	888	482	182 (169)	4.1 (4.1)	437 (359)	0.79 (0.79)
28%	45	2972	2480	492	238 (118)	3.5 (3.2)	371 (190)	0.64 (0.68)
	60	959	844	500	74 (74)	2.4 (2.4)	118 (118)	0.55 (0.55)
	80	4397	3950	505	122 (38)	2.2 (1.7)	203 (53)	0.47 (0.46)
	107	2482	1824	503	86 (52)	2.6 (2.4)	110 (91)	0.58 (0.61)
	142	3408	2938	500	94 (51)	2.3 (2.4)	127 (68)	0.50 (0.62)
	0	1478	1047	484	134 (112)	3.9 (3.7)	205 (155)	0.79 (0.79)
	10	1635	1250	478	218 (165)	4.4 (4.2)	405 (307)	0.81 (0.83)
	20	2202	1658	481	246 (155)	4.4 (4.2)	468 (243)	0.79 (0.83)
	30	6191	4611	479	465 (152)	4.6 (3.9)	702 (350)	0.75 (0.78)
32%	45	1853	1542	488	301 (218)	4.3 (4.4)	584 (489)	0.76 (0.81)
	60	3401	2477	479	309 (160)	4.4 (4.0)	451 (239)	0.77 (0.80)
	80	1195	1007	480	197 (165)	4.3 (4.2)	370 (398)	0.82 (0.82)
	107	1167	853	476	163 (158)	4.1 (4.1)	267 (247)	0.80 (0.80)
	142	2530	2149	485	259 (152)	4.2 (3.8)	423 (339)	0.75 (0.75)

Abbreviation: OTU, operational taxonomic unit.

* The numbers of the high quality reads were normalized to 844 reads and OUT numbers and diversity indices from the original and normalized (in parentheses) sequencing reads were calculated by the RDP pyrosequencing pipeline.

### Bacterial successions in saeu-jeot samples with different salt concentrations

**Figure 3:**
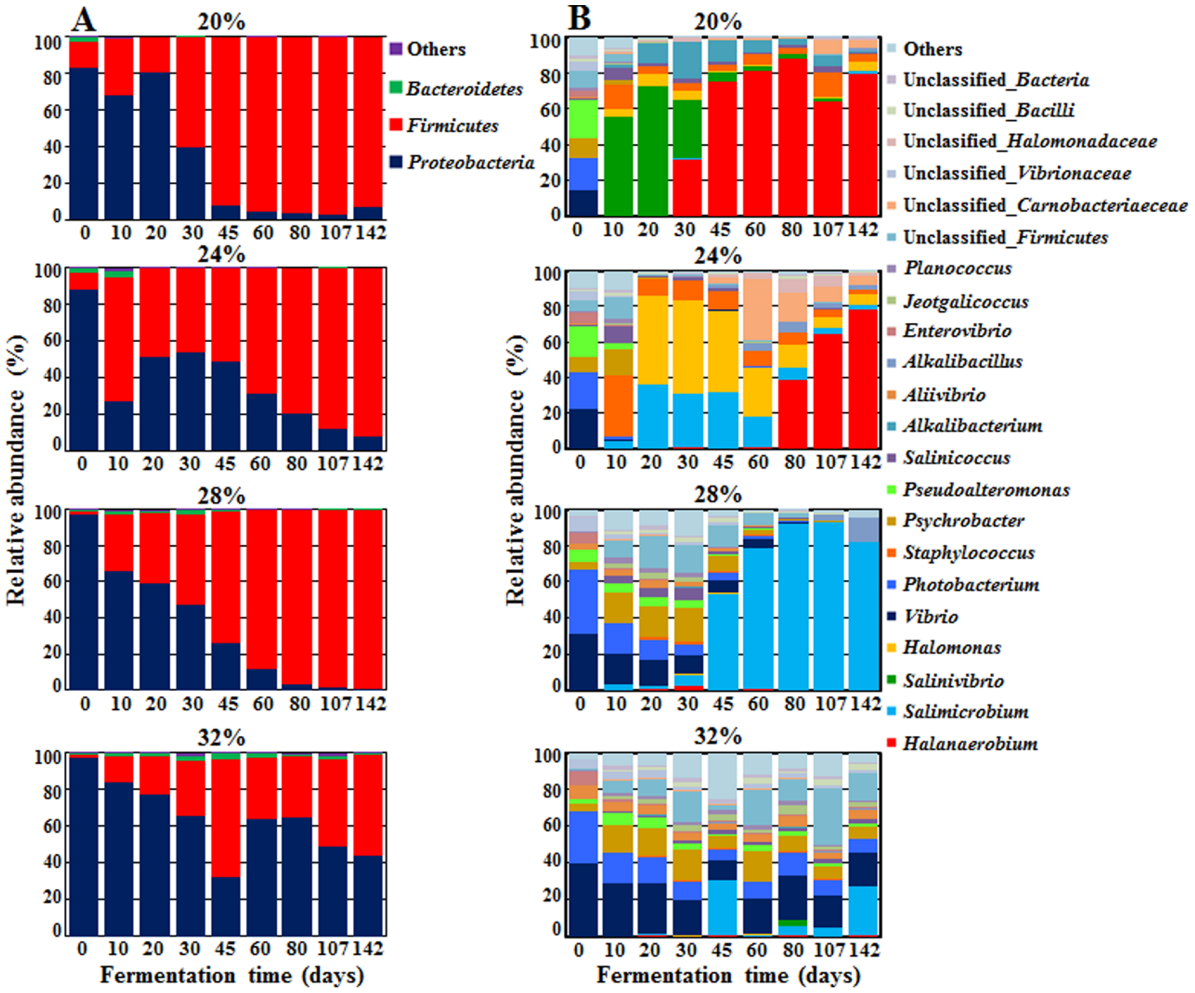
Bacterial taxonomic compositions showing the bacterial successions of saeu-jeot samples with 20%, 24%, 28%, and 32% salt concentrations at the phylum (A) and genus levels during saeu-jeot fermentation. Others are composed of the phyla or the genera, each showing a percentage of reads <3.0% of the total reads in all samples of each panel.

The bacterial sequencing reads were classified at the phylum and genus levels (Fig. 3). At the phylum level, the phyla *Proteobacteria* and *Firmicutes* were predominant in all saeu-jeot samples (Fig. 3A). *Proteobacteria* was predominant in the initial samples, but was rapidly replaced with *Firmicutes* as the fermentation progressed, as reported previously [8], [9]. The bacterial succession generally occurred more rapidly under low salt conditions than under high salt conditions. *Firmicutes* predominated, with more than 93% abundance after only 45 days in the 20% salted samples, whereas *Proteobacteria* remained as a dominant phylum until the end of fermentation in the 32% salted samples. However, at day 10, *Firmicutes* increased more quickly with a decrease of *Proteobacteria* in the 24% salted samples than in the 20% salted samples, even though *Proteobacteria* was maintained abundantly in the 24% salted samples until the late fermentation period.

The genus level analysis showed that *Vibrio*, *Photobacterium*, *Phychrobacter*, *Enterovibrio*, and *Pseudoalteromonas* belonging to *Proteobacteria* were dominant in the initial saeu-jeot samples, but the bacterial successions were significantly different depending on the salt concentrations (Fig. 3B). In the 20% salted samples, the initially dominant genera disappeared within only 10 days, and *Salinivibrio* became the most prevalent genus, followed by *Staphylococcus*, and eventually *Halanaerobium* predominated during the late fermentation period. The growth of *Salimicrobium*, which was a predominant genus in other salt concentrations and in other studies [8], [9], was not observed in the 20% salted samples. In the 24% salted samples, the initially dominant genera also disappeared quickly and *Staphylococcus* (of the *Firmicutes*) became predominant, explaining the rapid decrease of *Proteobacteria* at 10 day sample in the phylum level. Members of *Halomonas* belonging to *Proteobacteria* were predominant after 20 days and remained even until the late fermentation period, which explained the high abundance of *Proteobacteria* during the late fermentation period compared to the 20% salted samples (Fig. 3A). Eventually, *Halanaerobium* became predominant during the late fermentation period, similar to the 20% salted samples. In the 28% salted samples, the initially dominant genera, except for *Phychrobacter*, gradually decreased as the fermentation progressed; *Salimicrobium* became predominant after 45 days (Fig. 3B). In the 32% salted samples, the initially dominant genera remained until the end of the fermentation and the bacterial community was not predominated by particular genera, which might suggest that *Bacteria* did not grow well in 32% salted samples. In the 28% and 32% salted samples, the growth of *Salinivibrio*, *Staphylococcus*, *Halomonas*, and *Halanaerobium*, which were predominant in the 20% and 24% salted samples, was not observed.

**Figure 4:**
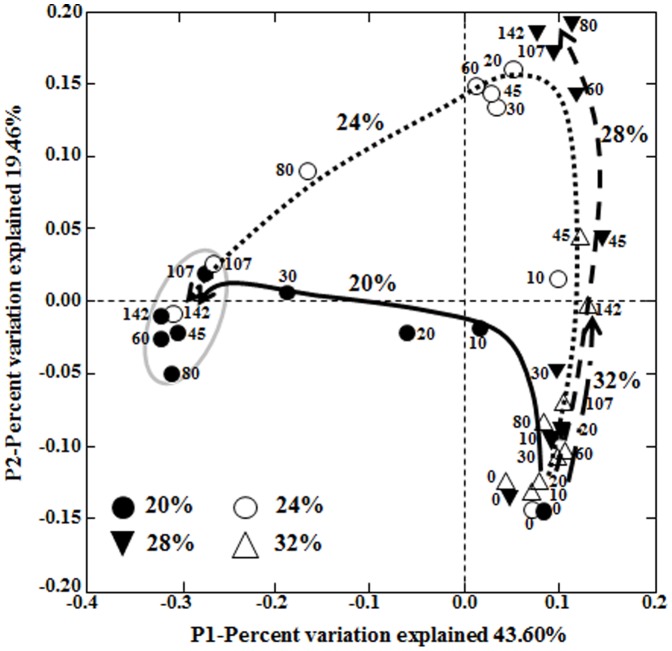
The weighted principle coordinate analysis (PCoA) plot showing the bacterial successions in saeu-jeot samples with 20%, 24%, 28%, and 32% salt concentrations during saeu-jeot fermentation. Numbers beside the symbols represent the fermentation time (days). The curved arrows indicate the routes of the bacterial successions on the PCoA plot during saeu-jeot fermentation. Saeu-jeot samples inside the circle included *Halanaerobium* as the predominant genus.

PCoA also supported the successional patterns of *Bacteria* (Fig. 4). The bacterial successions in the 20% and 24% salted samples occurred quite differently from 10 days of fermentation, but eventually the bacterial communities became similar during the late fermentation period. Bacterial successions in the 28% and 32% salted samples occurred similarly, but the bacterial succession in the 28% salted samples occurred more rapidly than in the 32% salted samples. Because it has been demonstrated that community analyses can be biased by noises associated with singletons [46], PCoA was also performed using the sequence data sets without singletons representing 0.6∼10.0% of the high quality reads; the analysis showed that the presence of singletons did not affect the PCoA results (data not shown).

### Metabolite changes in saeu-jeot samples with different salt concentrations

**Figure 5:**
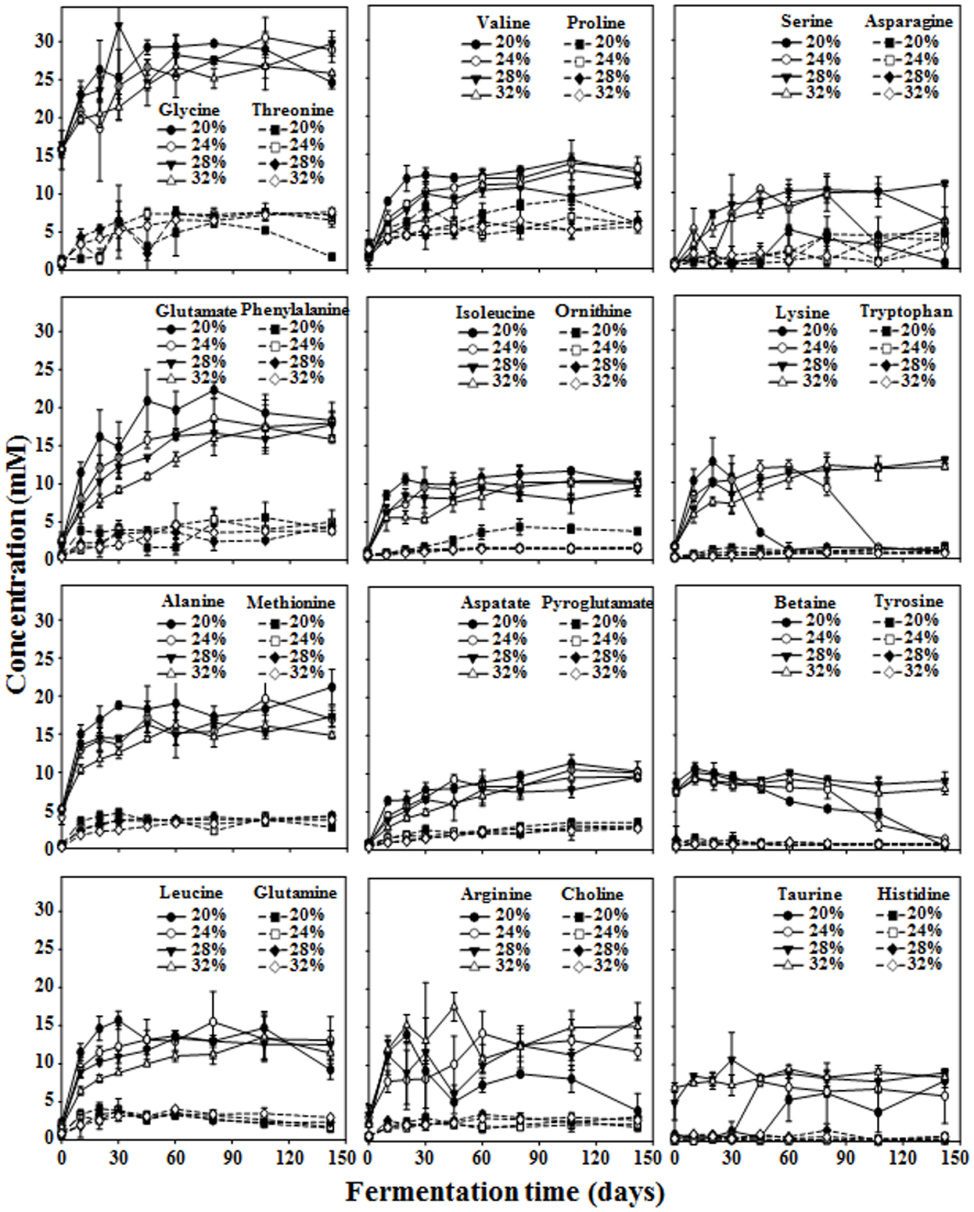
Changes in the major amino acids and nitrogen compounds identified from saeu-jeot samples with 20%, 24%, 28%, and 32% salt concentrations during saeu-jeot fermentation. Data are presented as average values ± standard deviations, measured in triplicate.

A ^1^H NMR technique was applied to analyze metabolites such as amino acids, nitrogen compounds, organic acids, and methylamines in saeu-jeot samples. Interestingly, the concentrations of amino acids increased in all saeu-jeot samples as the fermentation progressed regardless of salt concentrations (Fig. 5). However, the concentration-fermentation time profiles of some amino acids and nitrogen compounds were a little different depending on the salt concentrations. The concentrations of arginine and lysine increased more rapidly during the early fermentation period in the saeu-jeot samples with lower salt concentrations; however, after the rapid increase, the concentration of these amino acids decreased rapidly.

**Figure 6:**
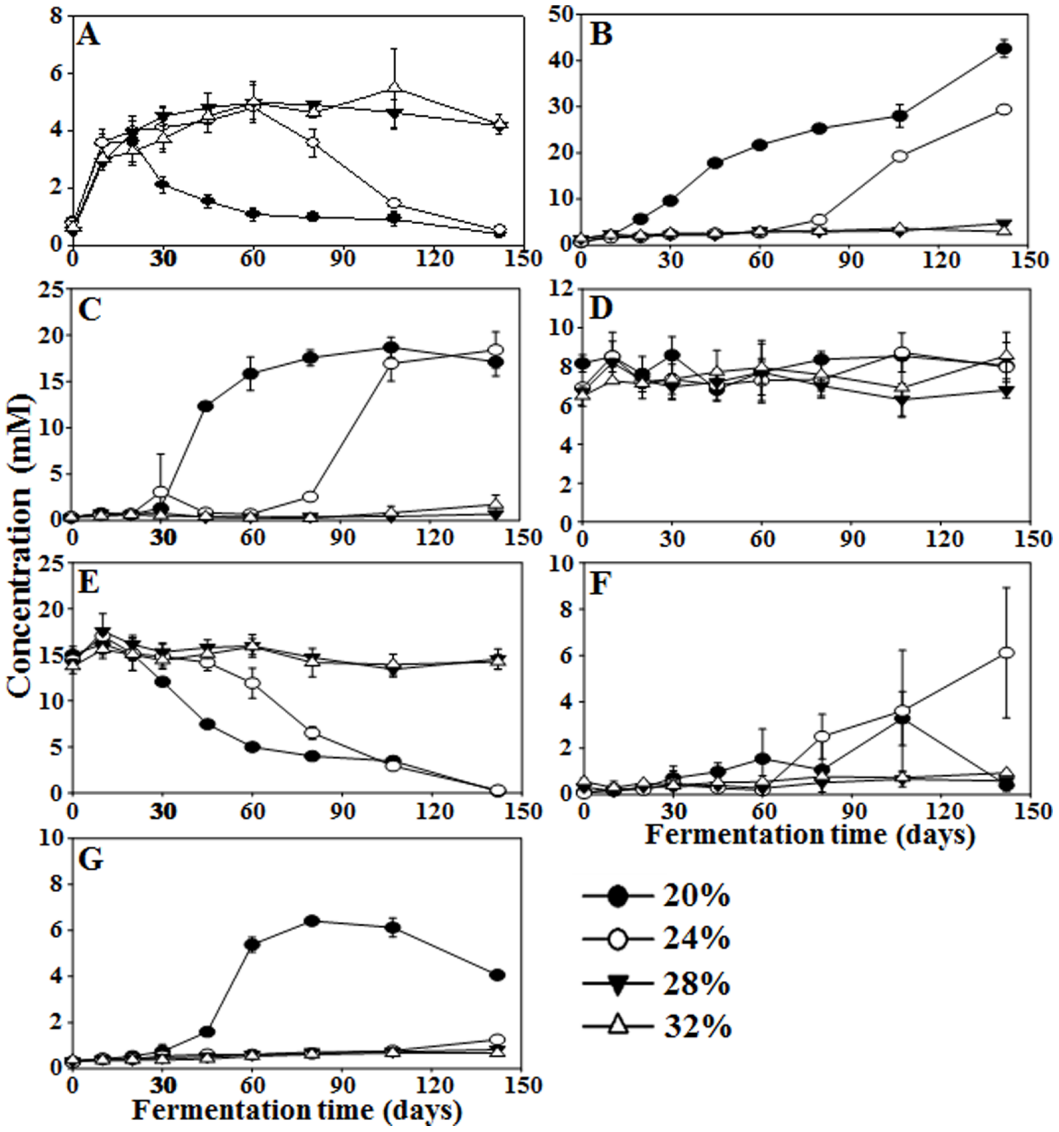
Changes in major organic compounds [glycerol (A), acetate (B), butyrate (C), and lactate (D)] and methylamines [trimethylamine *N*-oxide (E), trimethylamine (F), and dimethylamine (G)] identified from saeu-jeot samples with 20%, 24%, 28%, and 32% salt concentrations during saeu-jeot fermentation. Data are presented as average values ± standard deviations, measured in triplicate.

**Figure 7:**
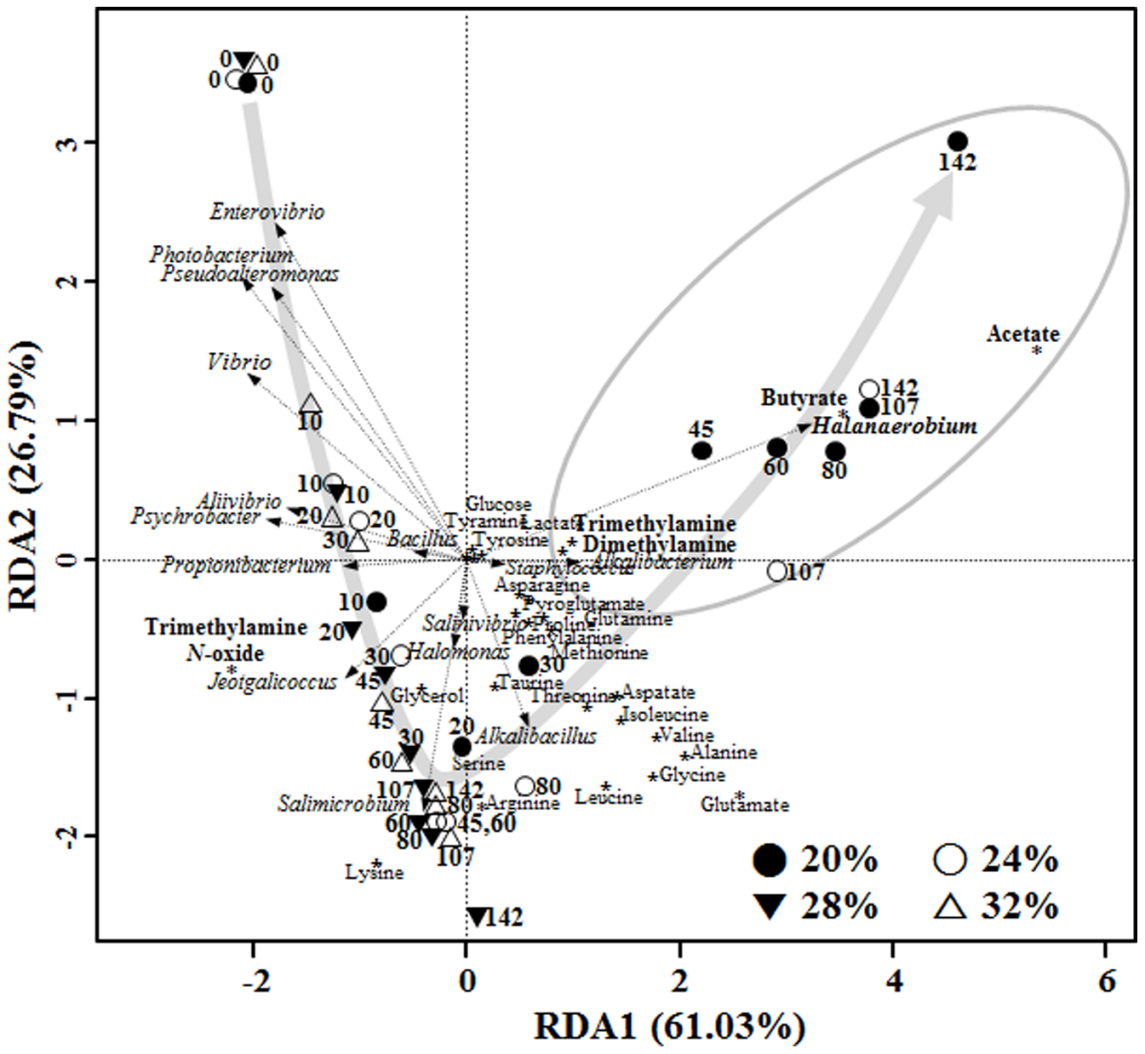
An RDA (redundancy analysis) triplot showing correlations among saeu-jeot samples, bacterial abundances, and metabolite concentrations during fermentation of saeu-jeot with 20%, 24%, 28%, and 32% salt concentrations. Numbers beside the symbols represent the fermentation time (days). The primary organic compounds and methylamines are highlighted in bold, and the directions and lengths of the straight arrows indicate their influences on the saeu-jeot samples. The curved arrow indicates the route of metabolite changes in saeu-jeot samples on the RDA triplot during saeu-jeot fermentation. Saeu-jeot samples inside the circle contained acetate, butyrate, TMA, and DMA as the major products.

Glycerol, acetate, butyrate, and lactate were identified as the primary carbonic compounds in saeu-jeot fermentation (Fig. 6). Interestingly, the glycerol level increased rapidly in all saeu-jeot samples, regardless of the salt concentrations, during the early fermentation period (Fig. 6A), and the glycerol level decreased quickly in the 20% and 24% salted samples after approximately 20 days and 60 days, respectively. However, in the 28% and 32% salted samples, the glycerol level was relatively constant until the end of fermentation. The levels of acetate and butyrate increased quickly in the 20% and 24% salted samples after approximately 20 days and 60 days, respectively (Figs. 6B and C), which was related to the glycerol decrease. However, in the 28% and 32% salted samples, the concentrations of acetate and butyrate were very low until the end of the fermentation. The lactate concentrations in all saeu-jeot samples were almost constant over the entire fermentation period (Fig. 6D). Methylamines, including trimethylamine (TMA) and dimethylamine (DMA), which cause the unique odors of fermented fish products, are generated by the reduction and/or demethylation of trimethylamine *N*-oxide (TMAO) [48]. In the 20% salted samples, TMAO rapidly decreased after approximately 20 days, with an increase in TMA and DMA (Figs. 6E, F, and G). In the 24% salted samples, TMAO also decreased with an increase in TMA, but an increase in DMA rarely occurred. However, in the 28% and 32% salted samples, the concentration of TMAO was almost constantly maintained, without the increase in TMA and DMA, until the end of the fermentation. To statistically assess the metabolite changes during the fermentation, RDA was performed on the basis of metabolites and bacterial abundances of the saeu-jeot samples (Fig. 7). The RDA triplot showed that the metabolite changes occurred similarly, with rapid rates during the early fermentation period, independent of the salt concentration. However, the RDA triplot also demonstrated that the metabolite profiles of the 20% and 24% salted samples during the late fermentation period were distinct from those of the other samples by the production of acetate, butyrate, dimethylamine, and trimethylamine, which were clearly related to the growth of *Halanaerobium* in the 20% and 24% saeu-jeot samples during the late fermentation stage (Fig. 7).

## Discussion

It is generally thought that *Bacteria* and *Archaea* play important roles in jeotgal fermentation because jeotgal is fermented under highly salted [20–32% (w/w)] conditions [3]. Isolation of novel archaeal species from fermented jeotgal (http://bacterio.net) [7] and functional roles of archaeal members such as histamine degradation and improved organoleptic acceptance in salted fermented seafood [49], [50] have been also reported. However, it was recently reported that *Archaea* are much less important than *Bacteria* in jeotgal fermentation [8], [9]. Our qPCR analysis also showed that *Bacteria* were more abundant than *Archaea* in all saeu-jeot samples, for all salt concentrations (Figs. 1B and C), suggesting that bacterial members may make a greater contribution to jeotgal fermentation than archaeal members even in high salt conditions (28% and 32%). However, in highly salted saeu-jeot samples the archaeal abundance increased, whereas the bacterial abundance decreased. This observation suggests that *Archaea* may play more important roles in highly salted saeu-jeot than in lower salted saeu-jeot.

Community analysis was performed for only *Bacteria* because *Bacteria* were in greater abundance than *Archaea* in all salted samples. Bacterial community and metabolite analyses showed that the growth of *Halanaerobium*, observed only in the 20% and 24% salted samples during the late saeu-jeot fermentation period, was associated with the metabolism of glycerol and TMAO as well as the production of acetate, butyrate, TMA, and DMA as reported previously (Figs. 3B, 4, 6, and 7) [8], [9]. *Halanaerobium* is a potential indicator for the putrefaction or over-fermentation of seafood by the production of acetate, butyrate, and methylamines [8], [9], [51]. Because *Halanaerobium* became predominant after 30–80 days of fermentation in the 20% and 24% salted samples (Fig. 3B), saeu-jeot fermentation should be stopped within 30–80 days to produce good quality saeu-jeot. In the 28% salted samples, the initially dominant genera *Vibrio*, *Photobacterium*, *Psychrobacter*, *Pseudoalteromonas*, and *Enterovibrio*, which may include potentially pathogenic strains [52], decreased as the fermentation progressed. *Salimicrobium*, known as a genus of non-pathogenic bacteria, predominated during the late fermentation period (Fig. 3B). During the late fermentation period, the bacterial diversity was quite low (the asymptotes nearly converged, Fig. 2C), suggesting that *Salimicrobium* predominated, with the extinction of initially dominant genera. However, in the 32% salted samples, the initially dominant genera still remained as dominant genera, even until the end of fermentation (Fig. 3B). These results suggest that 32% salt concentration may not be appropriate for the production of safe saeu-jeot.

The generation of amino acids from proteins by proteolysis during seafood fermentation is important because it influences taste and flavor [30], [53]–[55]. The concentration of amino acids steadily increased until approximately 80–107 days of the saeu-jeot fermentation at all salt concentrations, which indicated that the concentration of amino acids affecting saeu-jeot taste in the 20% salted samples increased continually, even after the appearance of *Halanaerobium* (30 days) and the production of acetate, butyrate, and methylamines. These results suggest that saeu-jeot with 20% salt concentration may not be appropriate in view of the production of good quality and tasty saeu-jeot. The fermentation of the 24% salted saeu-jeot might be stopped within 80 days before *Halanaerobium* dominated, while the fermentation of the 28% salted saeu-jeot might be progressed at least for 80 days, maximizing amino acid concentrations, until the initially dominant genera disappeared. These results suggest that a salt concentration in the range of 24–28% in saeu-jeot fermentation may be appropriate for the production of safe and tasty saeu-jeot based on the bacterial community and metabolite composition.

Interestingly, our metabolite analysis showed that the profiles of amino acids were relatively similar during the entire saeu-jeot fermentation period, regardless of the salt concentrations. However, the bacterial growth and successions were considerably different depending on the salt concentration, suggesting that proteolysis to amino acids might not be related to the bacterial population (Figs. 1B and 5). Previous results also showed that bacterial proteinases isolated from saeu-jeot had very weak proteinase activities under high salt conditions [5], [56]. Besides the concentrations of the amino acids, the level of glycerol, which might be derived from lipid hydrolysis, also increased with a similar rate regardless of the salt concentrations during the early fermentation period (Fig. 6A), which indicated that the increase might not be related to the bacterial population. Diverse endogenous enzymes such as carboxypeptidase A and B, chymotrypsin, cathepsin, and lipase have been identified from shrimp or fish [22], [57]–[59], but the activities of the endogenous enzymes have not been tested under high salt conditions. Therefore, further studies will be necessary to investigate whether these endogenous enzymes are responsible for the increase in amino acids and glycerol during the saeu-jeot fermentation.

To the best of our knowledge, this was the first study to investigate the effects of salt concentration on the microbial succession and metabolite changes during saeu-jeot fermentation. However, additional studies of the relationship among microbial communities, metabolites, sensory characteristics (e.g., taste and flavor), and food safety are necessary to produce high quality and tasty saeu-jeot.

## Supporting Information

Table S1 List of adapter and barcode sequences in the PCR primer sets used in this study.
(DOCX)
