# Supplementary material for: Microbial Successions and Metabolite Changes during Fermentation of Salted Shrimp (Saeu-Jeot) with Different Salt Concentrations
Source: PLoS One. 2014 Feb 28;9(2):e90115. doi: 10.1371/journal.pone.0090115 (PMC3938600; doi:10.1371/journal.pone.0090115)
Supplement: Table S1 — List of adapter and barcode sequences in the PCR primer sets used in this study. (DOCX) [file pone.0090115.s001.docx]

**Supporting Information**

**Table S1** List of adapter and barcode sequences in the PCR primer sets used in this study

| Subject | | Sequence (5'-3') | Reference |
| --- | --- | --- | --- |
| Salt conc. | Days |  |  |
| 20% | 0 | TATGCAC | This study |
|  | 10 | ACTGAGT |  |
|  | 20 | ATGCAGAC |  |
|  | 30 | CTGTGAT |  |
|  | 45 | TCTGCAG |  |
|  | 60 | AGCGATG |  |
|  | 80 | CACACTGA |  |
|  | 107 | ATCGTCTGTG |  |
|  | 142 | ATCGTAGCAG |  |
| 24% | 0 | TCATATACGCG | This study |
|  | 10 | TAGATAGTGCG |  |
|  | 20 | AGATCGCT |  |
|  | 30 | TCGCTATC |  |
|  | 45 | CAGTCTCGA |  |
|  | 60 | AGTCACTAG |  |
|  | 80 | TGAGTGACGC |  |
|  | 107 | TCTGTCTCGC |  |
|  | 142 | TCGCAGACAC |  |
| 28% | 0 | TCAGATG | This study |
|  | 10 | CGATGAG |  |
|  | 20 | TACAGCAG |  |
|  | 30 | ACTACACGC |  |
|  | 45 | TGACTCGAC |  |
|  | 60 | CTGTCTACG |  |
|  | 80 | ACGTCTCTACG |  |
|  | 107 | ATACACGAGCG |  |
|  | 142 | ACACACGCATC |  |
| 32% | 0 | TCGTCAT | This study |
|  | 10 | ATGCTGAG |  |
|  | 20 | AGAGCTG |  |
|  | 30 | ACTGAGT |  |
|  | 45 | ACTCGTATC |  |
|  | 60 | CGTGTACTG |  |
|  | 80 | CACACGATAG |  |
|  | 107 | ATGTGTCTAG |  |
|  | 142 | ATGTACGATG |  |
| Adapter sequence | |  |  |
| A adapter | | CCATCTCATCCCTGCGTGTCTCCGACTCAG | Roesch et al. (2007) |
| B adapter | | CCTATCCCCTGTGTGCCTTGGCAGTCTCAG |  |

**Reference**

1. Roesch LF, Fulthorpe RR, Riva A, Casella G, Hadwin AK, et al. (2007) Pyrosequencing enumerates and contrasts soil microbial diversity. ISME J 1: 283-290. doi:10.1038/ismej.2007. PubMed: 53 18043639.
